# Breastfeeding rates in England during the Covid-19 pandemic and the previous decade: Analysis of national surveys and routine data

**DOI:** 10.1371/journal.pone.0291907

**Published:** 2023-10-11

**Authors:** Maria A. Quigley, Sian Harrison, Ilana Levene, Jenny McLeish, Phyll Buchanan, Fiona Alderdice

**Affiliations:** 1 NIHR Policy Research Unit in Maternal and Neonatal Health and Care, National Perinatal Epidemiology Unit, Nuffield Department of Population Health, University of Oxford, Oxford, United Kingdom; 2 National Perinatal Epidemiology Unit, Nuffield Department of Population Health, University of Oxford, Oxford, United Kingdom; 3 Breastfeeding Supporter, The Breastfeeding Network, Paisley, United Kingdom; Menzies School of Health Research, AUSTRALIA

## Abstract

**Background:**

Few studies have compared breastfeeding rates before and during the pandemic using comparable data across time. We used data from two national maternity surveys (NMS) to compare breastfeeding rates in England before and during the pandemic.

**Methods:**

Analysis was conducted using the NMS from 2018 (pre-pandemic; n = 4,509) and 2020 (during the pandemic; n = 4,611). The prevalence of breastfeeding initiation, and ‘any’ breastfeeding and exclusive breastfeeding (EBF) at 6 weeks and 6 months were compared between these surveys. Data were interpreted in the context of underlying trends in these prevalences from previous NMS (from 2010 and 2014), and annual routine data for England (from 2009–10 to 2020–21). Modified Poisson regression was used to estimate adjusted risk ratios (aRR) for the effect of birth during the pandemic (2020 versus 2018) on breastfeeding, with adjustment for sociodemographic and birth-related factors.

**Results:**

Breastfeeding initiation and any breastfeeding at 6 weeks remained relatively constant in the NMS and the routine data. Birth during the pandemic was associated with a 3 percentage point decrease in EBF at 6 weeks in the NMS (aRR 0.92, 95%CI: 0.87, 0.98 for pandemic versus pre-pandemic), but a smaller decrease in the routine data. Birth during the pandemic was associated with a 3 percentage point increase in any breastfeeding at 6 months in the NMS (aRR 1.05, 95%CI: 1.00, 1.10). Breastfeeding varied across different groups of women in the NMS (i.e. marked inequalities), but the small changes observed between the pandemic and pre-pandemic NMS were broadly similar across the sociodemographic and birth-related factors examined (i.e. no change in inequalities).

**Conclusion:**

Breastfeeding initiation and any breastfeeding at 6 weeks in England were unaffected by the pandemic, and the persistent inequalities in breastfeeding did not widen. Services should aim to reduce these inequalities in breastfeeding which have been documented since the 1970s.

## Introduction

Current national and international guidance recommends breastfeeding for most babies and this advice remained unchanged during the COVID-19 pandemic [1, 2]. The pandemic disrupted maternity care in the UK, with many routine maternity and breastfeeding services being cancelled, reduced or delivered virtually [3–5]. The pandemic also resulted in increased separation of mothers and babies in hospital, reduced contact with professional and social support networks, exclusion of birth partners from appointments and births, and increased stress and anxiety [3, 4, 6, 7]. Taken together, these factors are likely to have had a detrimental effect on breastfeeding rates and women’s experiences of breastfeeding during the pandemic.

Many women have reported that their breastfeeding experience was adversely affected by the additional challenges of giving birth during the pandemic [5]. A recent review of disruption to services caused by COVID-19 in five Western countries, including the UK, found that women commonly reported insufficient support to enable them to continue breastfeeding [8]. Some women, however, have reported a more positive experience because increased time at home and fewer visitors made it easier to bond with the baby and focus on breastfeeding [5, 8]. Therefore, COVID-19 and the consequent changes and restrictions may have served as an additional barrier to breastfeeding for some women but may have facilitated breastfeeding for others.

It is important to compare breastfeeding rates before and during the pandemic to assess the impact of COVID-19 and the disruption to services and support that ensued. Furthermore, assessment of how the known barriers and facilitators of breastfeeding, such as sociodemographic and birth-related factors, affected breastfeeding rates, and applying knowledge gained from the pandemic-based alterations in access to, and provision of, breastfeeding services will inform maternity care and relevant policies.

National routine data on breastfeeding initiation and breastfeeding at 6–8 weeks in England have been published annually since 2003–4 and 2007–8 respectively. However, national or area-based aggregate statistics can mask variations in prevalence according to different sociodemographic and birth-related characteristics. As the impacts of COVID-19 were unevenly distributed across different geographical areas and sociodemographic groups [9], it is important to assess the impact of COVID-19 on breastfeeding practices for different groups of women.

Population-based national maternity surveys (NMS) have been carried out regularly by the National Perinatal Epidemiology Unit (NPEU) since 1995. The two most recent surveys were of women giving birth in May 2020 (during the COVID-19 pandemic) and women giving birth in October 2017 (pre-pandemic). We used data from these two surveys to compare breastfeeding rates in England before and during the pandemic (objective 1) and to determine whether any changes observed in breastfeeding rates remained after adjusting for sociodemographic and birth-related factors (objective 2). We hypothesised that changes in breastfeeding rates during the pandemic may have varied according to different sociodemographic and birth-related factors, and therefore we tested for this (objective 3). Our results were interpreted in the context of underlying trends in breastfeeding rates during 2010–20 based on previous NMS and routine data for England.

## Methods

### Design and participants

The NMS in 2018 [10] and 2020 [6] were cross-sectional postal and online surveys. For each survey, random population-based samples of approximately 16,000 women were identified by the Office for National Statistics (ONS) using birth registration records. The women were aged 16 years or older, living in England at the time the birth was registered, and had given birth to their baby in England during a two-week period in October 2017 (in the 2018 NMS) or in May 2020 (in the 2020 NMS). In both surveys, women were invited to take part six months after they had given birth.

The response rate to the surveys was 28% in 2018 (n = 4,509) and 29% in 2020 (n = 4,611). Some anonymised sociodemographic information was provided for all women (including non-responders) by ONS. In both surveys, comparison of responders and non-responders showed that response was lower in younger women, unmarried women, women born outside the UK, women living in disadvantaged areas and women who had given birth previously [6, 10]. We attempted to correct for these differences using survey weights which were derived using maternal age, marital/registration status, whether born outside the UK, region of residence, area deprivation (measured by the Index of Multiple Deprivation, IMD), and parity.

The questionnaires for both surveys had a similar format and covered similar topics. Women self-reported sociodemographic characteristics and maternity experiences during pregnancy, birth, and the postnatal period, and there were detailed sections about infant feeding.

Both surveys were performed in accordance with all relevant guidelines and regulations. Ethical approval was obtained from the London Bloomsbury NRES Committee (REC reference 18/LO/0271) for the 2018 survey on 22^nd^ February 2018 and from the North West—Greater Manchester East NRES Committee (REC reference 20/NW/0426) for the 2020 survey on 22^nd^ October 2020. These committees waived the requirement for informed consent. Participants were provided with a study information sheet that included all information typically included in a consent form and could contact the study team to discuss the study. Return of partially or fully completed questionnaires was considered to imply agreement to participate in the surveys and consent to use the data.

### Breastfeeding outcomes

The prevalence of several breastfeeding outcomes was estimated: breastfeeding initiation (i.e. the proportion of all women who ever gave breastmilk to their baby); and ‘any’ breastfeeding and exclusive breastfeeding (EBF) at 6 weeks and 6 months (i.e. the proportion of all women who were breastfeeding at these time-points). EBF was based on questions about the last time the baby received breastmilk and the first time the baby received formula, other liquids, or solids. Prevalence at six weeks was chosen for consistency with national statistics on breastfeeding at 6–8 weeks. Prevalence at six months was chosen for consistency with the recommended guidelines [11, 12] and also because most babies were at least six months old at the time of our surveys.

### Statistical analysis

The prevalence of the breastfeeding outcomes was estimated for the 2018 NMS (pre-pandemic) and the 2020 NMS (during the pandemic). In order to interpret the data in the context of underlying trends in breastfeeding, these prevalences were plotted alongside estimates from other relevant data sources related to the same or previous time periods. First, we analysed data on breastfeeding initiation and breastfeeding at 6 weeks from previous NMS (2010, 2014). These surveys had a similar study design to the later NMS except that women were surveyed at 3 rather than 6 months [13]. Second, we used published estimates for breastfeeding initiation and breastfeeding at 6–8 weeks based on national routine data for the years from April 2009-March 2010 to April 2020-March 2021, where the latter year was the first year of the pandemic. These data were collected from Primary Care Trusts until April 2013, since when they have been collected from maternity services providers and child health information systems. Aggregate data for England and by ‘area’ (previously Primary Care Trust, currently Local Authority) have been reported by various government arms-length bodies [14, 15]. Data on breastfeeding at 6 months are not collected routinely and were not collected in the NMS prior to 2018.

For the main study objectives, data from the 2018 and 2020 NMS were combined, and survey year was fitted as the main explanatory factor in the regression models, thus giving an estimate for the effect of birth during the pandemic on breastfeeding rates. As the study outcomes were common, modified Poisson regression [16] was used to estimate unadjusted (objective 1) and adjusted (objective 2) risk ratios (RRs) for survey year.

In the adjusted models, the explanatory factors were selected *a priori* as being strongly associated with breastfeeding in previous UK studies [17], and were included in the models irrespective of p-values. These explanatory factors were maternal age, age left full-time education, IMD, ethnicity, whether born outside the UK, parity, mode of birth, hospital length of stay, preterm birth and whether the baby had a neonatal admission (see variable categories in Table 1). Finally, we tested whether the observed changes in breastfeeding (i.e. RRs for survey year) varied according to the other explanatory factors (objective 3). Here, the regression models included interaction terms between survey year and each explanatory factor (shown in Table 1) in turn. Stratum-specific RRs were presented if the interaction was statistically significant (p<0.05).

**Table 1:** Characteristics of the women included in the two surveys for the analysis of breastfeeding initiation and EBF.

	BF initiation		EBF 6 weeks	
	Pre-pandemic (2018 NMS)	During pandemic (2020 NMS)	Pre-pandemic (2018 NMS)	During pandemic (2020 NMS)
	N = 4,155	N = 4,611	N = 4,509	N = 4,611
	n (%)#	n (%)#	n (%)#	n (%)#
Age				
<25	323 (13.8)	296 (12.4)	312 (14.0)	289 (12.5)
25–34	2,438 (58.6)	2,560 (59.5)	2,328 (58.4)	2,473 (59.5)
35+	1,394 (27.6)	1,346 (28.1)	1,327 (27.6)	1,293 (28.0)
Age left fulltime education				
< = 16	450 (14.4)	466 (13.6)	432 (14.5)	448 (13.6)
17–18	973 (26.2)	1,123 (28.4)	937 (26.4)	1,093 (28.8)
19+	2,732 (59.4)	2,613 (58.0)	2,598 (59.1)	2,514 (57.6)
IMD				
5 richest	929 (16.0)	921 (15.7)	880 (15.9)	891 (15.8)
4	942 (17.7)	982 (18.2)	908 (18.0)	953 (18.3)
3	876 (18.9)	880 (19.5)	835 (18.9)	849 (19.6)
2	797 (22.1)	800 (21.6)	762 (22.3)	767 (21.4)
1 poorest	611 (25.3)	619 (24.9)	582 (25.0)	595 (24.9)
Ethnicity				
White British	3,171 (71.0)	3,244 (69.1)	3,042 (71.6)	3,145 (69.6)
White other	462 (11.6)	411 (13.7)	443 (11.7)	398 (13.7)
Mixed	94 (2.6)	92 (2.5)	87 (2.6)	88 (2.5)
Asian	246 (8.2)	305 (8.6)	229 (8.1)	285 (8.4)
Black	87 (3.6)	99 (4.5)	76 (3.1)	92 (4.4)
Other	95 (2.9)	51 (1.6)	90 (2.9)	47 (1.5)
Born outside UK	876 (26.3)	793 (28.3)	829 (25.9)	748 (27.6)
Nulliparous	2,210 (45.5)	2,183 (46.4)	2,113 (45.5)	2,112 (46.5)
Caesarean birth	1,211 (27.2)	1,315 (30.2)	1,147 (28.9)	1,264 (30.3)
Length of stay >24 hours	2,160 (49.8)	1773 (43.5)	2,034 (49.0)	1,706 (42.4)
Preterm birth	292 (7.5)	289 (7.5)	278 (7.4)	272 (7.2)
Neonatal admission	489 (11.7)	425 (10.7)	465 (11.7)	406 (10.5)

*These numbers show those with any missing explanatory factors, after excluding those with missing outcome data

^#^All counts are unweighted and percentages are weighted

All analysis was conducted in Stata version 17 (StataCorp. 2021), using survey-weighted commands to allow for non-response. There was very little missing data for the breastfeeding outcomes (S1 Table), being around 1% for all outcomes except EBF which was missing for 5% of women because it required recorded answers to several questions. Around 8% of women had missing data on explanatory factors (S1 Table), and a complete case analysis was employed on the remaining 92% of women (range 88–92%).

## Results

### Characteristics of the women included in the pre-pandemic and pandemic NMS

Data were available on 9,120 women (4,509 from the 2018 NMS; 4,611 from the 2020 NMS). The number of women included in the analysis of each outcome varied (S1 Table). Table 1 shows the characteristics of the women included in the analysis of breastfeeding initiation (which had the largest sample size) and EBF at 6 weeks/months (which had the smallest sample size). The distribution of the characteristics was broadly similar across outcomes and surveys, the largest difference between surveys being a shorter length of stay in hospital in the pandemic. All differences were adjusted for in the analysis.

### Changes in breastfeeding before and during the pandemic

There was no change during the pandemic in the proportion of women who initiated breastfeeding (remaining at 85%) or who were breastfeeding at 6 weeks (around 64%) (Table 2). The proportion of women who were breastfeeding at 6 months increased slightly during the pandemic from 45.1% to 48.3% (adjusted RR 1.05, 95% CI: 1.00, 1.10, p = 0.046). In contrast, the proportion of women who were EBF at 6 weeks decreased slightly during the pandemic from 40.9% to 38.2% and this difference was statistically significant (aRR 0.92, 95% CI: 0.87, 0.98, p = 0.006). However, the reverse happened at 6 months, with the proportion of women EBF increasing (from 16.0% to 18.0%), although after adjustment this difference was not statistically significant (aRR 1.10, 95% CI: 0.99, 1.21, p = 0.070).

**Table 2:** RRs for the effect of survey year on the outcomes.

	Pre-pandemic (2018 NMS)		During pandemic (2020 NMS)		During vs pre-pandemic	
					Crude RR	Adjusted#
	N	%	N	%	(95% CI)	RR (95% CI)
Breastfeeding initiation	4,155	85.3	4,202	84.7	0.99 (0.97, 1.02)	0.99 (0.97, 1.01)
					P = 0.55	P = 0.44
Breastfeeding at 6 weeks	4,126	64.0	4,182	64.6	1.01 (0.97, 1.05)	1.01 (0.97, 1.04)
					P = 0.57	P = 0.76
Breastfeeding at 6 months	4,126	45.1	4,182	48.3	1.07 (1.02, 1.13)	1.05 (1.00, 1.10)
					P = 0.009	P = 0.046
EBF at 6 weeks	3,967	40.9	4,055	38.2	0.93 (0.88, 0.99)	0.92 (0.87, 0.98)
					P = 0.028	P = 0.006
EBF at 6 months	3,967	16.0	4,055	18.0	1.12 (1.01, 1.25)	1.10 (0.99,1.21)
					P = 0.027	P = 0.070

^#^ Adjusted for maternal age, age left full-time education, IMD, ethnicity, born outside the UK, parity, mode of birth, hospital length of stay, preterm birth and neonatal admission

**Fig 1:**
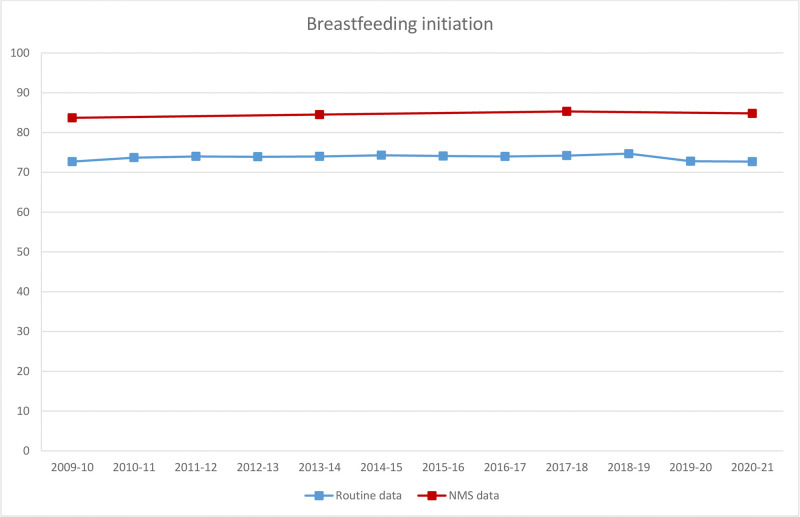
Breastfeeding initiation by year of birth across the NMS and routine data for England. Note: Women gave birth in 2009 and the NMS was conducted in 2010; women gave birth in 2017 and the NMS was conducted in 2018; weights could not be calculated for the 2010 NMS.

**Fig 2:**
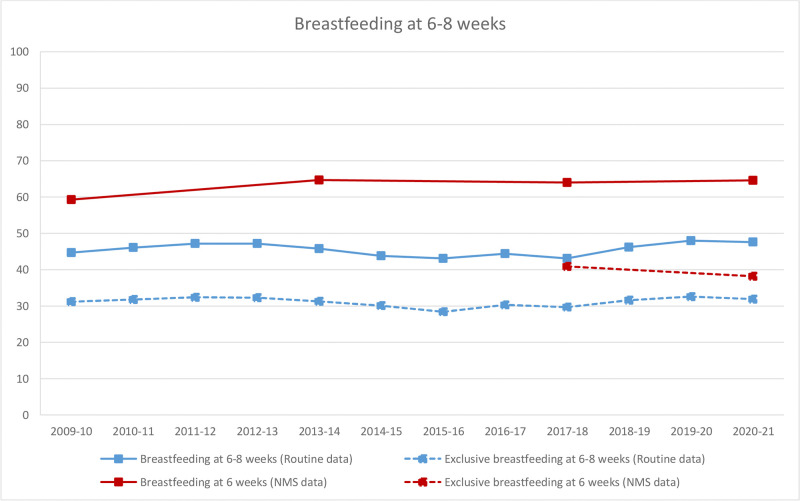
Breastfeeding at 6–8 weeks by year of birth across the NMS and routine data for England. Note: Women gave birth in 2009 and the NMS was conducted in 2010; women gave birth in 2017 and the NMS was conducted in 2018; weights could not be calculated for the 2010 NMS.

These pandemic and pre-pandemic estimates were plotted alongside other estimates from 2010–2020. The prevalence of both breastfeeding initiation (Fig 1) and breastfeeding at 6 weeks (Fig 2) was consistently higher in the NMS than the routine data although the trends were broadly similar. For breastfeeding initiation, the prevalence remained relatively constant in 2010–20 in both data sources, with no large change observed in the routine data in the pandemic (April 2020-March 2021). For breastfeeding at 6 weeks, the prevalence has remained constant in the NMS since 2014. For any breastfeeding and EBF at 6–8 weeks (Fig 2), the routine data showed a small drop in prevalence in the pandemic (April 2020-March 2021), but this change was no larger than the year-on-year fluctuations observed during the previous decade, with EBF closely tracking any breastfeeding.

### Did the changes in breastfeeding vary according to other factors?

S1–S5 Figs show the crude prevalence of the breastfeeding outcomes by survey year and other selected sociodemographic and birth–related explanatory factors. Breastfeeding initiation varied according to most of the explanatory factors explored, although the patterns were similar before and during the pandemic (S1 Fig). There were particularly strong patterns across the sociodemographic factors, with women the least likely to breastfeed if they were younger, with lower levels of education, living in more socially deprived areas, of white British ethnicity and born in the UK. The same patterns were observed for any breastfeeding at 6 weeks and 6 months, and EBF at 6 weeks and 6 months (S2–S5 Figs). Importantly these patterns were identical before and during the pandemic. The only exception was that preterm babies born in the pandemic tended to have lower rates of breastfeeding compared to preterm babies born before the pandemic.

When we formally tested whether the adjusted RRs for the pandemic varied according to the explanatory factors (objective 3), there was no strong evidence of interaction (S2 Table), thus indicating that the changes observed during the pandemic (i.e. the aRRs) were broadly similar across the explanatory factors examined and certain groups were not affected disproportionately.

**Fig 3:**
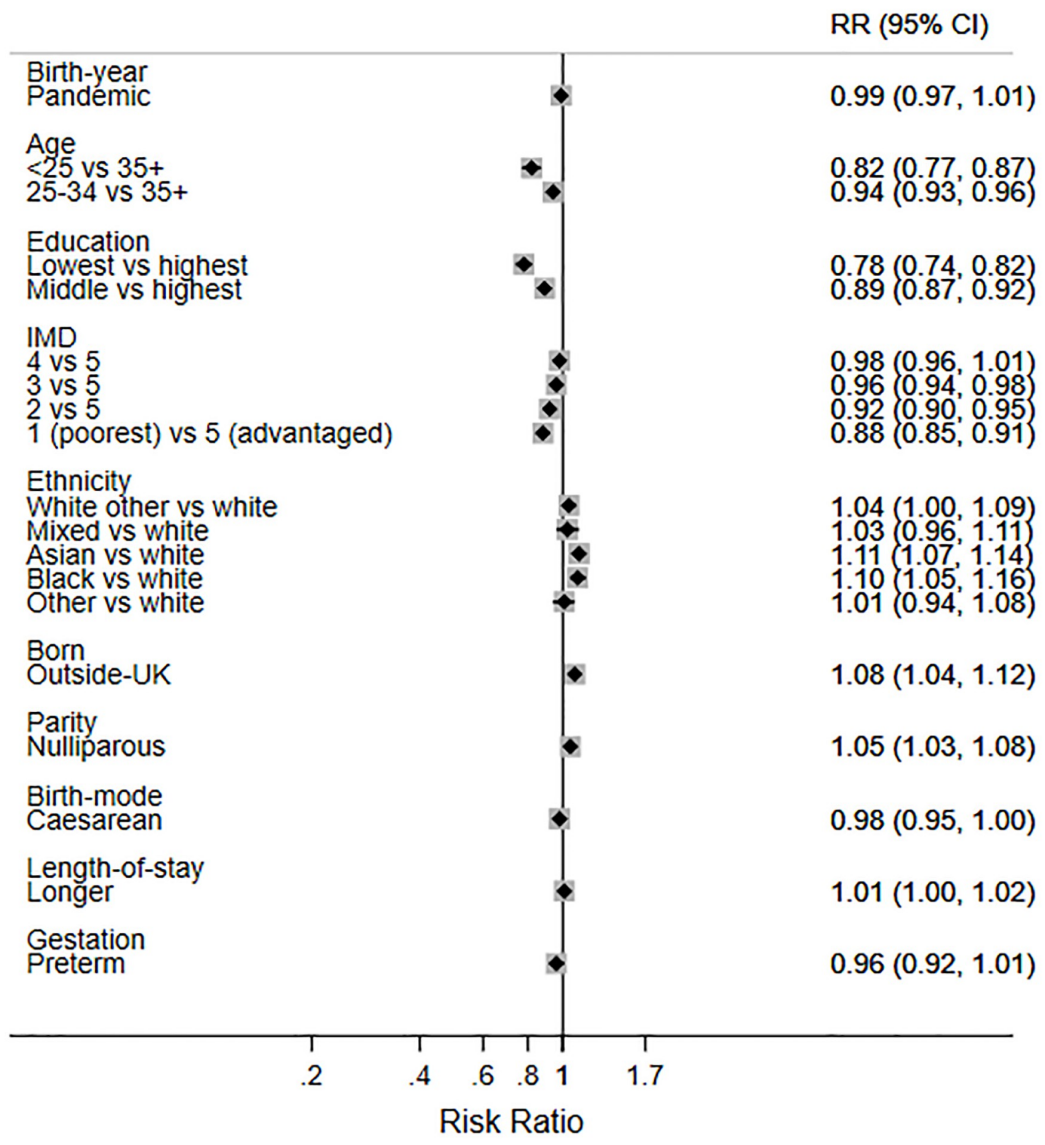
Adjusted RRs for breastfeeding initiation in 2018 and 2020 combined.

**Fig 4:**
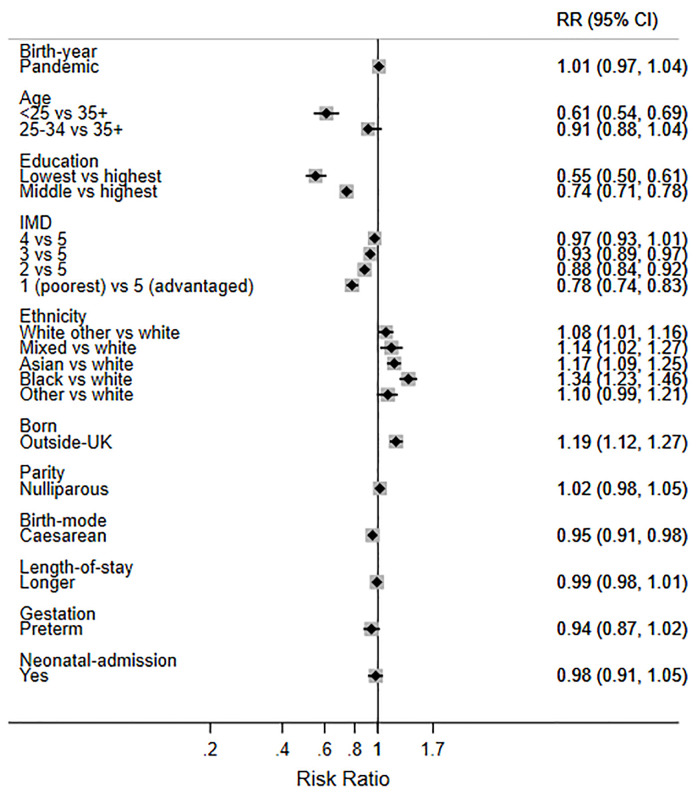
Adjusted RRs for any breastfeeding at 6 weeks in 2018 and 2020 combined.

**Fig 5:**
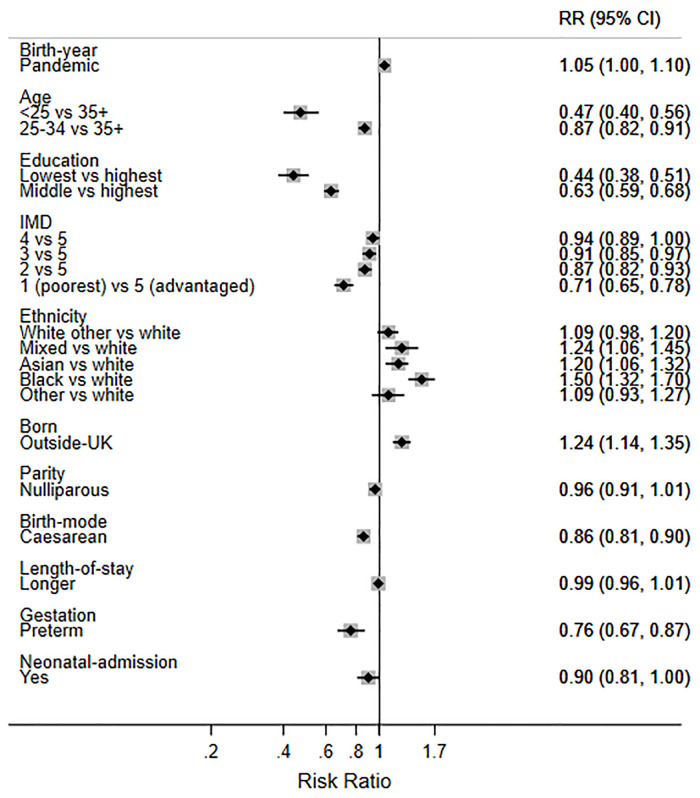
Adjusted RRs for any breastfeeding at 6 months in 2018 and 2020 combined.

**Fig 6:**
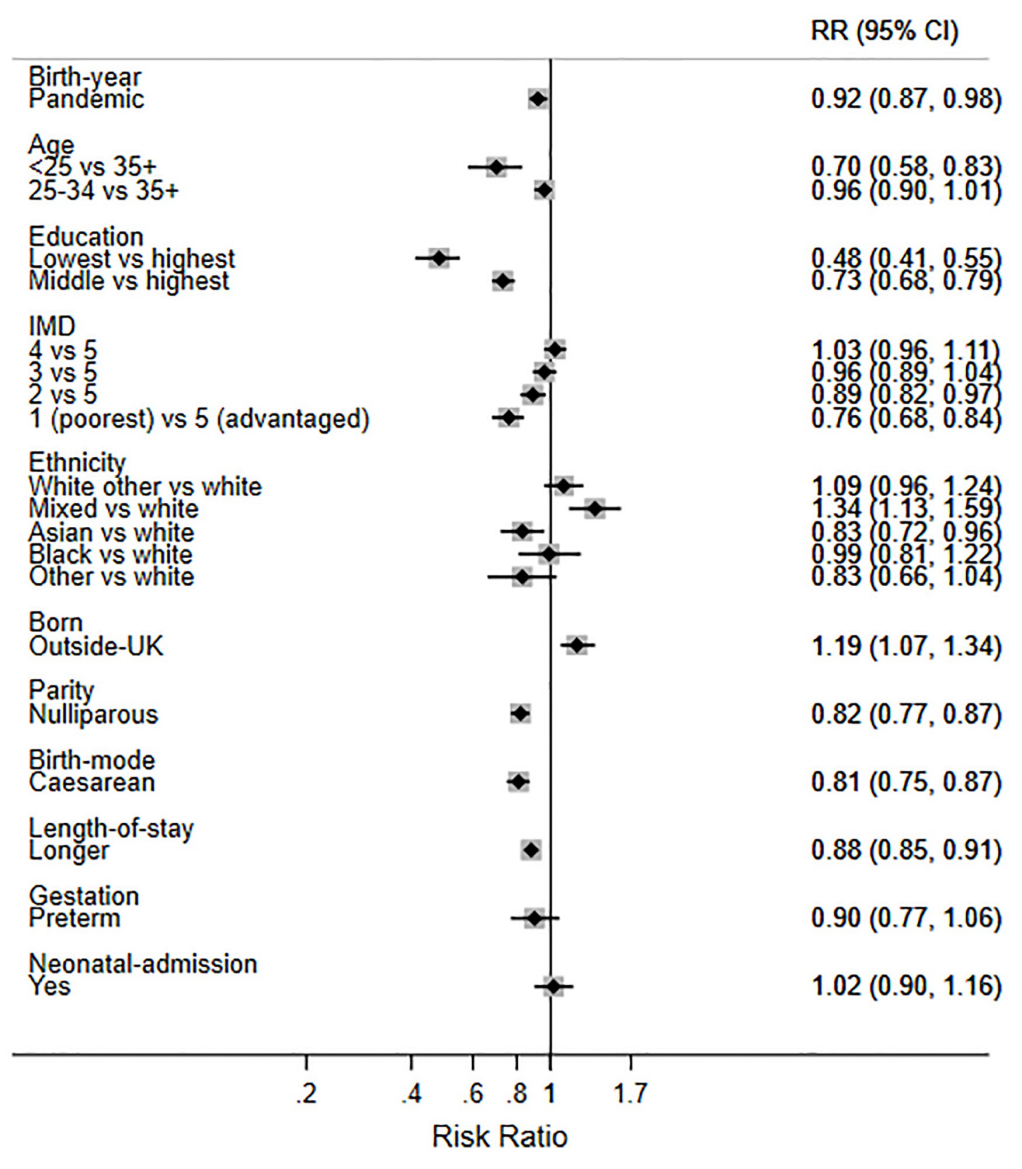
Adjusted RRs for exclusive breastfeeding (EBF) at 6 weeks in 2018 and 2020 combined.

**Fig 7:**
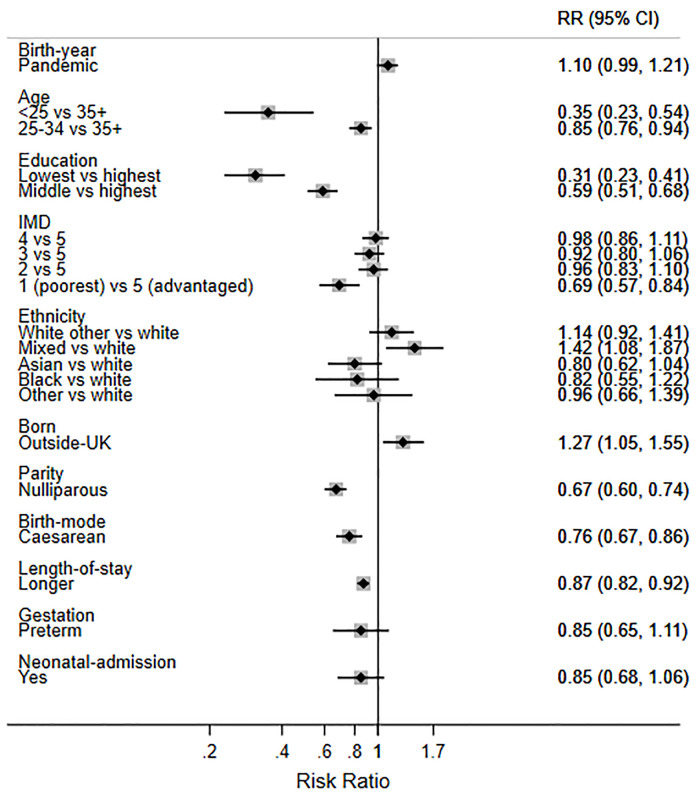
Adjusted RRs for exclusive breastfeeding (EBF) at 6 months in 2018 and 2020 combined.

Finally, in order to help interpret the size of the two statistically significant changes observed during the pandemic, we present the mutually adjusted RRs for birth during the pandemic and the other factors (Figs 3–7). While birth during the pandemic was associated with an 8% decrease in EBF at 6 weeks (aRR 0.92) and a 5% increase in any breastfeeding at 6 months (aRR 1.05), other factors were much stronger determinants of these outcomes. For example, the women least likely to EBF at 6 weeks were under 25 years (aRR 0.70), less educated (aRR 0.48), living in deprived areas (aRR 0.76), nulliparous (aRR 0.82), and had a caesarean birth (0.81) (Fig 6). Compared with White British women, Asian women were less likely to EBF at 6 weeks (aRR 0.83), while women of mixed heritage (aRR1.34) and those born outside the UK (aRR 1.19) were more likely to EBF at 6 weeks.

## Discussion

### Summary

Breastfeeding initiation and any breastfeeding at 6 weeks in England remained relatively constant during the first year of the pandemic, in both the NMS and the routine data. However, giving birth during the pandemic was associated with a small decrease in EBF at 6–8 weeks in the routine data, which was consistent with the year-on-year fluctuations, whereas there was a 3 percentage point decrease in EBF at 6 weeks in the NMS. In contrast, giving birth during the pandemic was associated with a 3 percentage point increase in any breastfeeding at 6 months in the NMS, however, there were no comparison data at 6 months from previous NMS or routine data to confirm or refute this finding. While these population-based estimates of breastfeeding varied across different groups of women in the NMS, the small changes in breastfeeding between the pandemic and pre-pandemic NMS were broadly similar across the sociodemographic and birth-related factors examined.

### Comparison with other studies

Trends in breastfeeding rates from before and during the pandemic have not yet been widely reported. A review which included several ‘before and after’ studies mostly from the US reported that, with the exception of one study, breastfeeding initiation and duration remained stable [8]. Trends in breastfeeding rates in routinely collected data from other parts of the UK also suggest that the pandemic was not associated with a marked change in breastfeeding prevalence in Wales [18] (breastfeeding initiation and at 10 days, 6 weeks, 6 months), Scotland [19] (any breastfeeding and EBF at 6–8 weeks) or Northern Ireland [20] (breastfeeding initiation and at hospital discharge). In repeated cross-sectional surveys in the Mid-West of Ireland in 2001–2020, an area with traditionally low breastfeeding rates, there was also no change in breastfeeding initiation or at hospital discharge in 2020 compared with the previous years [21]. Data on admissions to UK neonatal units showed a decrease in breastfeeding at hospital discharge in moderate-to-late preterm infants, but an increase in full-term infants in April-June 2020 compared with December 2019 to February 2020 [22]. This is consistent with the lower prevalence of breastfeeding at 6 weeks observed in the NMS for preterm babies born in the pandemic compared with pre-pandemic.

### Interpretation of findings

Taken together, the evidence suggests that breastfeeding rates in England did not change substantially during the pandemic, which is surprising for two reasons. First, maternity and breastfeeding services in England underwent many changes during the pandemic, particularly in 2020. In a survey of health professionals in UK maternity services in May 2020—when the mothers in our pandemic survey gave birth—56% reported a reduction in postnatal appointments and 89% reported using remote consultation methods [3]. Many breastfeeding services were cancelled, reduced or delivered virtually [5, 6]. The pandemic also resulted in increased separation of mothers and babies in hospital, reduced contact with support networks, exclusion of birth partners from appointments and births, and increased anxiety [3, 4, 6]. These changes alone would be expected to have had a detrimental effect on breastfeeding. Second, several studies have reported that women wanted more support with breastfeeding in the pandemic [5, 6]. In the 2020 NMS, nearly half of women (46%) wanted more support with breastfeeding from a health professional, which was a very large increase compared with 30% in the 2018 and 2014 surveys [6].

One possible reason for observing relatively small changes in breastfeeding rates during the pandemic is that some of the negative effects of the pandemic may have been offset by other aspects of the pandemic that helped facilitate breastfeeding. For example, maternity staff in a Scottish study believed that restricting visiting on postnatal wards was beneficial for maternal wellbeing, rest, bonding and breastfeeding [23]. Following hospital discharge, lockdowns may have meant that women did not go outside the home as much, had fewer visitors, and therefore had more uninterrupted time at home [5, 23]. In addition, government guidance on working from home and the furlough scheme may have allowed some partners to be around more and for longer than in pre-pandemic times [5]. Finally, some women may have ordinarily stopped breastfeeding to return to work, but working from home or furlough schemes may have facilitated ongoing breastfeeding. Before the pandemic, returning to work was associated with shorter breastfeeding duration [24]. These facilitative factors may have offset the negative effects of the pandemic at an individual and/or population level.

A second explanation is that although there was reduced in-person breastfeeding support, some women may have been more likely to receive ‘remote’ support [6]. Remote support may be provided by health professionals, peer supporters or lay persons, often by telephone, SMS messages, video call, or social media. A potential advantage of remote support is that it may be easier to access and be available ‘out of hours’. Women also use the internet to gather information and advice about breastfeeding [25]. Remote support is not a substitute for in-person support [26] but it may be a useful addition to other sources for women who have the IT resources to access it [5]. A systematic review suggested that remote breastfeeding support was associated with higher rates of EBF at 3 months (but not at 4–8 weeks or 6 months), particularly when compared with no support [27].

Finally, it is possible that some women who experienced difficulties managed to continue breastfeeding despite the disruption to maternity care, overcoming the increased challenges and displaying resilience [28]. In the NMS, EBF at 6 weeks was lower in the pandemic, but there was no change in any breastfeeding at 6 weeks, and even an increase at 6 months. This may suggest that more women had difficulties with breastfeeding in the early weeks—perhaps when support was needed the most—which resulted in the introduction of formula, either temporarily or more long term, but that they also continued breastfeeding. While data on breastfeeding difficulties were not collected in the NMS, loss of full breastfeeding at 6 weeks may reflect unresolved feeding difficulties and maternal confidence [28, 29]. Routine data for England showed a 7.5% increase in emergency department attendance for feeding problems in infants during the pandemic, including a 12.8% increase for neonatal jaundice [30]. In addition, we observed an increase in the prevalence of postnatal anxiety and depression in the NMS [6] and similar increases have been found in other studies [31, 32]. Although increases in neonatal jaundice or postnatal anxiety and depression cannot be attributed to breastfeeding difficulties, it is possible that challenges with feeding may have contributed to these outcomes.

While the pandemic had only a marginal effect on breastfeeding rates generally, other factors were strongly associated with breastfeeding. Nulliparous women were more likely to initiate breastfeeding, but tended to have a shorter duration, compared with parous women. Women who had a caesarean section or a preterm birth tended to have a shorter breastfeeding duration. The strongest sociodemographic determinants of not initiating breastfeeding or having a shorter duration were young age, having lower levels of education, living in a deprived area, White British ethnicity and being born in the UK. These sociodemographic inequalities in breastfeeding rates have been documented in the UK since the 1970s [17, 33, 34]. Targeting these inequalities is likely to be an effective way to increase breastfeeding rates in the UK. It is somewhat reassuring that the inequalities did not increase during the pandemic, at least for the factors we examined. This is plausible since many of the changes that occurred during 2020, which may have been barriers to, or facilitators of, breastfeeding occurred across many sociodemographic groups. Examples include the disruption to maternity services, reduction in social support networks due to lockdowns, working from home (in 2020, this was more common in occupations requiring higher qualifications [35]) and furlough schemes (in 2020–21, these were more common in those with fewer qualifications or who worked in arts, entertainment and recreation, and hospitality [36]).

### Strengths and limitations

The main strength of our pandemic NMS is that it is population-based and, unlike many pandemic-related surveys, we have a directly relevant comparison group based on a similar pre-pandemic survey. Our surveys included women who gave birth in a particular two-week period, who were contacted six months postnatally, therefore ensuring more homogeneous groups of women than other surveys. We report data on several breastfeeding outcomes, including breastfeeding at 6 months, which is not collected routinely in England. We also collected data on many sociodemographic and birth-related factors, and allowed for these in the analysis. Finally, rather than relying wholly on a ‘before and after’ design to estimate the pandemic effect, we interpreted our findings in the context of underlying trends in breastfeeding rates from previous NMS and routine data.

The main limitation of the NMS is potential responder bias owing to the low response. Despite employing strategies aimed at increasing survey response rates in the 2018 and/or 2020 NMS, such as increasing the number of reminders [37], using a quick response (QR) code to enable faster access to the online version of the questionnaire [37], using a push-to-web design [38], and including a financial incentive, the response rate in the 2018 survey (28%) was lower than in the 2014 survey (47%), although it did not drop again in 2020 (29%). We compared socio-demographic characteristics of respondents and non-respondents, and used survey weights to correct for these differences, but the NMS breastfeeding prevalences likely over-estimate the true values. There are also known issues with data completeness and quality in the routine data. For example, initiation was based on around 95% of all births prior to 2016–17, but 41–82% of all births since then. Prevalence estimates for breastfeeding at 6–8 weeks from routine data are likely biased downwards because women with missing data (between 4% and 13% in our study period) are counted as not breastfeeding. An additional limitation of our study is the lack of more detailed data on women’s infant feeding behaviours, including barriers and facilitators to breastfeeding.

## Conclusions

The first year of the pandemic resulted in significant changes to maternity services, and many aspects of life, but it appears that breastfeeding rates at the population level were not substantially affected by this. There was some evidence of a decrease in EBF at 6 weeks, but an increase in any breastfeeding at 6 months, perhaps reflecting a complex picture of breastfeeding barriers and facilitators at the population and individual level. While breastfeeding rates varied across sociodemographic and birth-related factors, these inequalities did not increase compared with those observed pre-pandemic. Although the number of women who breastfed changed very little during the pandemic, the numbers do not necessarily reflect quality of care or breastfeeding experiences more generally. Pregnancy and the postnatal period is a vulnerable time for women, and effective infant feeding support, with flexibility to access help when needed, should be available. Services should aim to reduce the stark and persistent inequalities in breastfeeding which have been documented since the 1970s.

## Supporting information

S1 Fig Proportion of women ever breastfed by survey year and other explanatory factors. (TIF)

S2 Fig Proportion of women breastfeeding at 6 weeks by survey year and other explanatory factors. (TIF)

S3 Fig Proportion of women breastfeeding at 6 months by survey year and other explanatory factors. (TIF)

S4 Fig Proportion of women exclusively breastfeeding at 6 weeks by survey year and other explanatory factors. (TIF)

S5 Fig Proportion of women exclusively breastfeeding at 6 months by survey year and other explanatory factors. (TIF)

S1 Table Missing data and sample size for complete case analysis by outcome. (DOCX)

S2 Table P-values for tests of interaction between survey year and each explanatory factor in turn. (DOCX)
