# Supplementary figures and images for: Breastfeeding rates in England during the Covid-19 pandemic and the previous decade: Analysis of national surveys and routine data
Source: PLoS One. 2023 Oct 11;18(10):e0291907. doi: 10.1371/journal.pone.0291907 (PMC10566678; doi:10.1371/journal.pone.0291907)

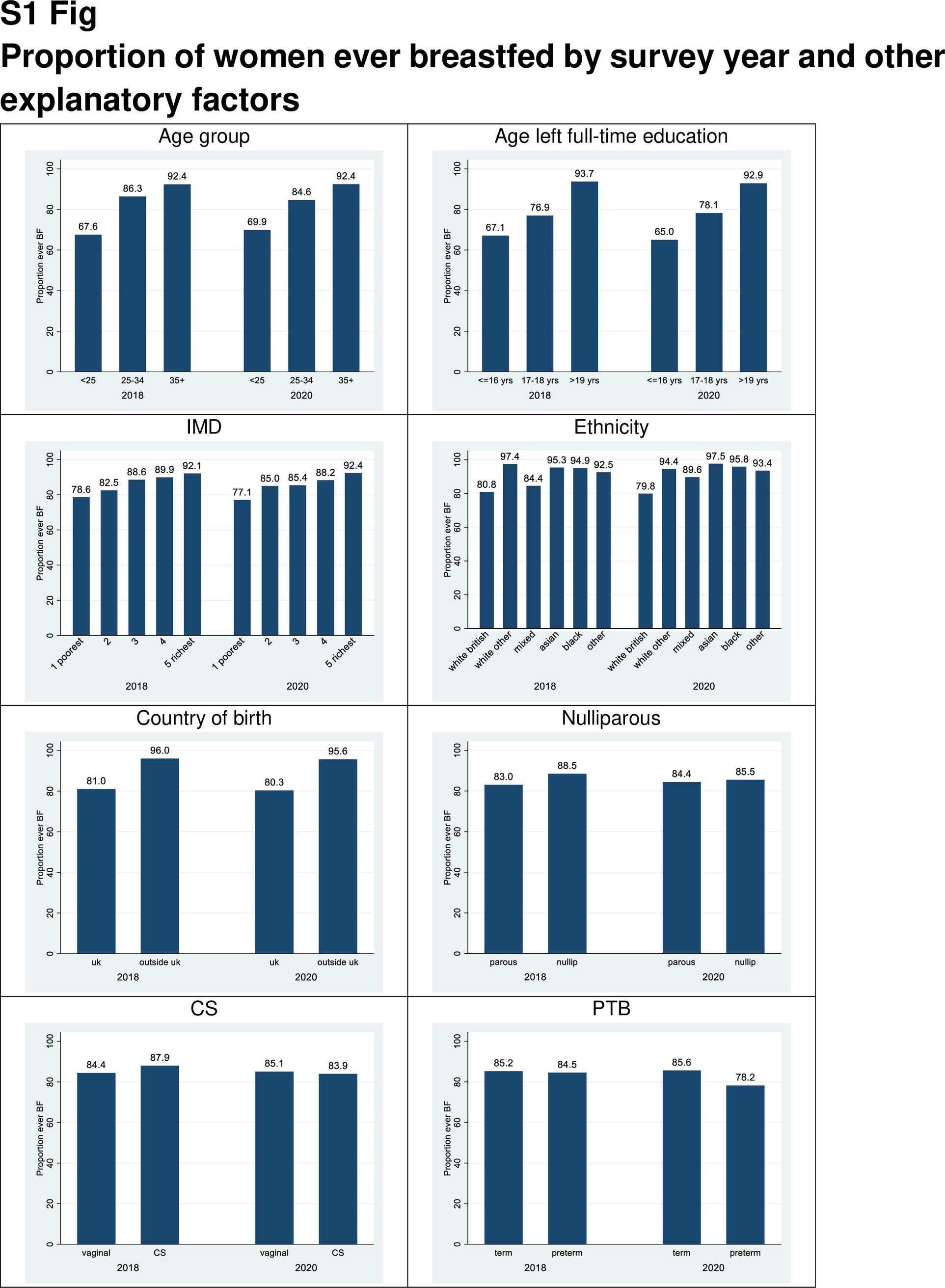

Supplement: S1 Fig — (TIF) [file pone.0291907.s001.tif]

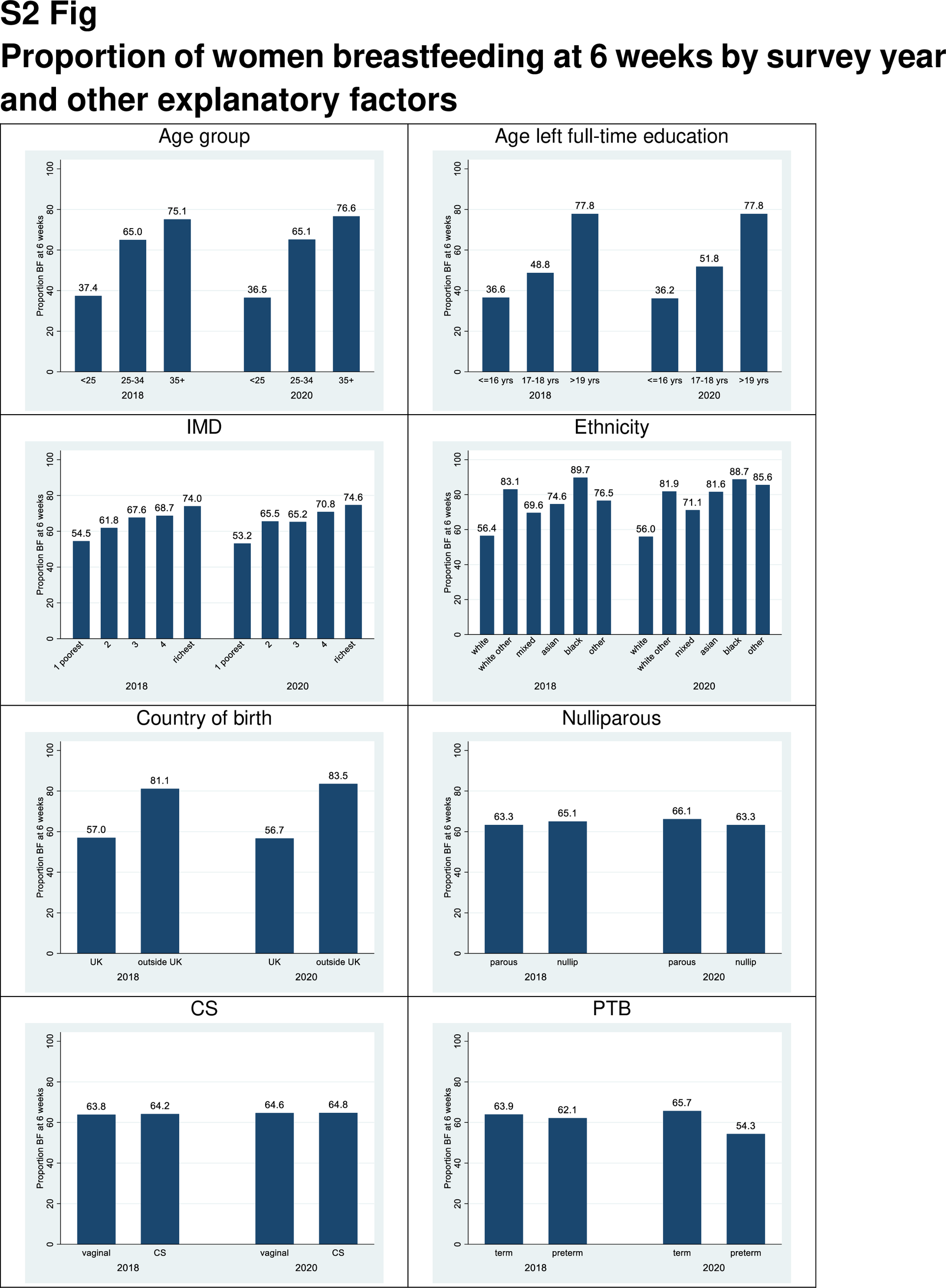

Supplement: S2 Fig — (TIF) [file pone.0291907.s002.tif]

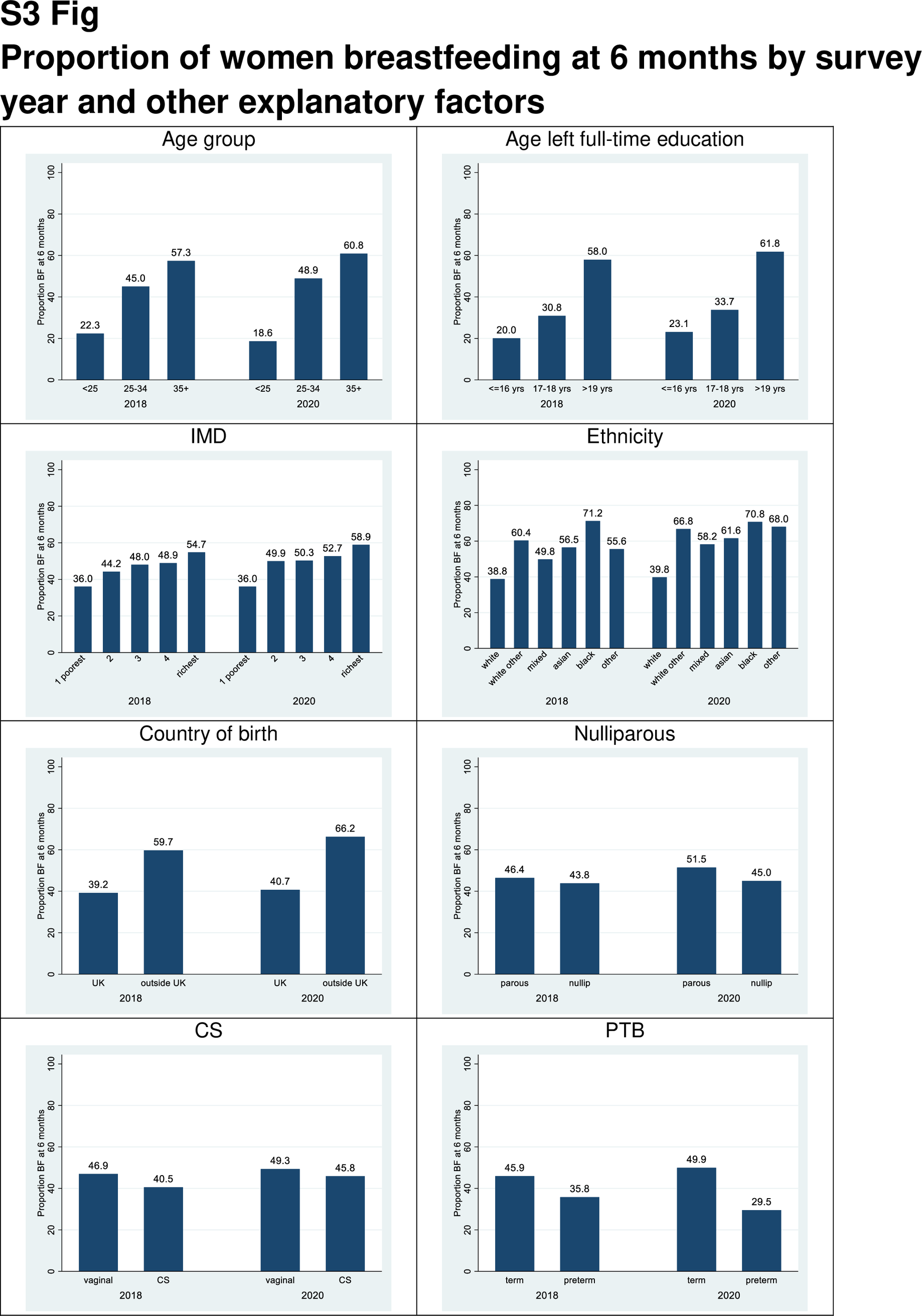

Supplement: S3 Fig — (TIF) [file pone.0291907.s003.tif]

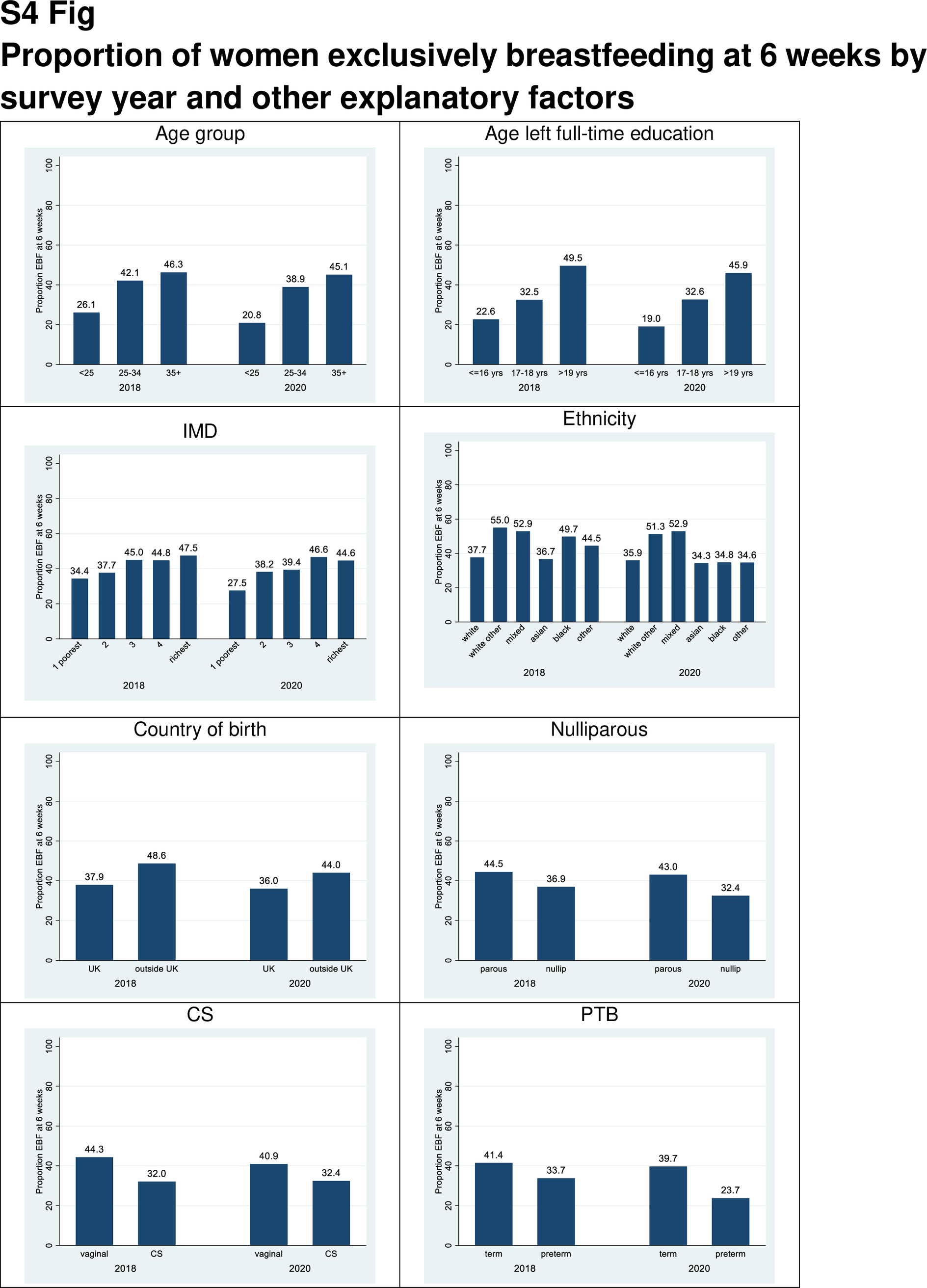

Supplement: S4 Fig — (TIF) [file pone.0291907.s004.tif]

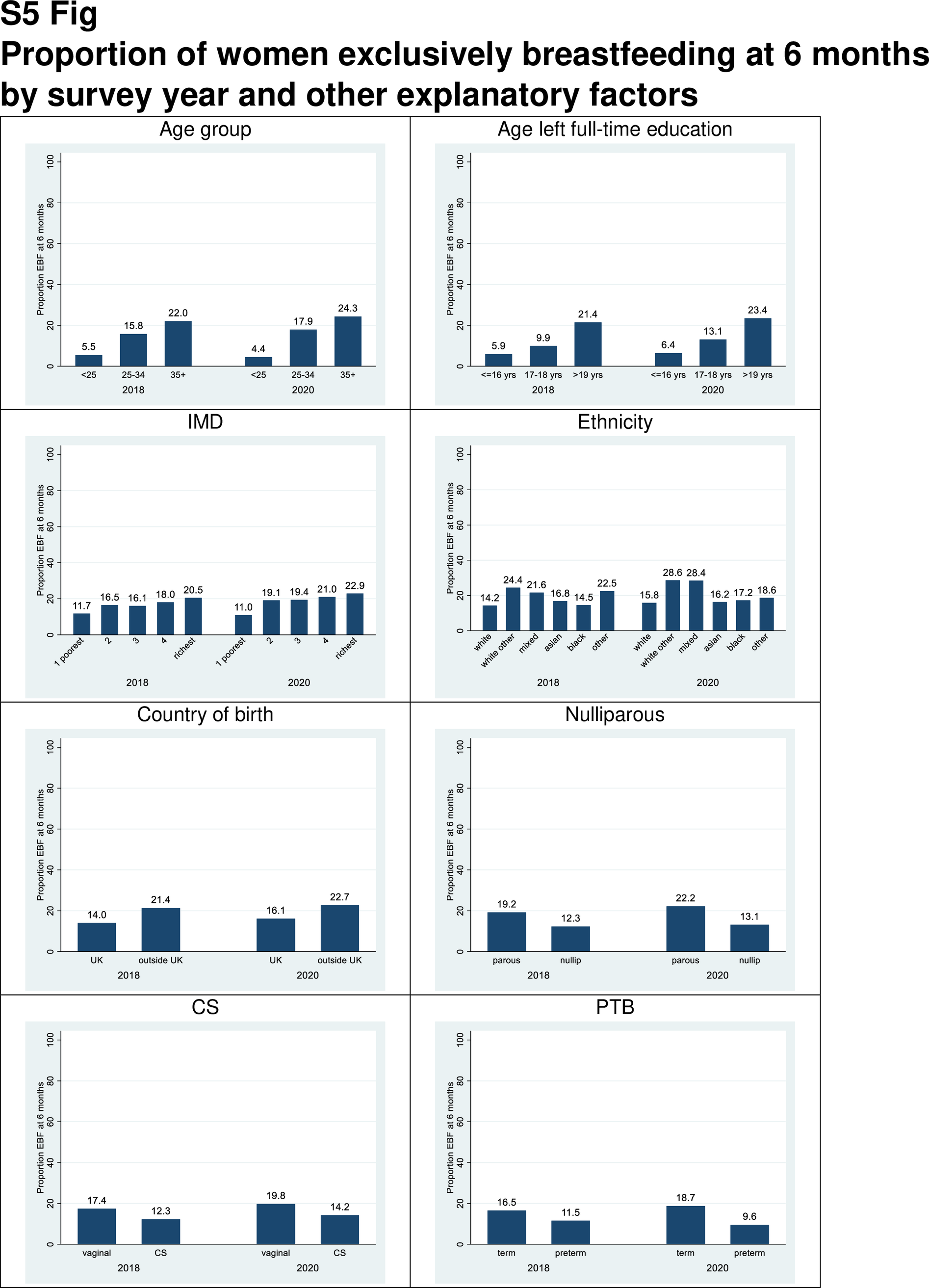

Supplement: S5 Fig — (TIF) [file pone.0291907.s005.tif]
