# Supplementary material for: Breastfeeding rates in England during the Covid-19 pandemic and the previous decade: Analysis of national surveys and routine data
Source: PLoS One. 2023 Oct 11;18(10):e0291907. doi: 10.1371/journal.pone.0291907 (PMC10566678; doi:10.1371/journal.pone.0291907)
Supplement: S1 Table — (DOCX) [file pone.0291907.s006.docx]

**S1 Table**

**Missing data and sample size for complete case analysis by outcome**

|  | **Pre-pandemic (2018 NMS)** | **During pandemic**  **(2020 NMS)** | Combined |
| --- | --- | --- | --- |
| Target | 16,000 | 16,050 |  |
| Responders | 4,509 | 4,611 | 9,120 |
| BF initiation  Missing outcome  Missing explanatory factors#  Complete case analysis | 13 (0.3)  341 (7.6)  4155 | 5 (0.1)  404 (8.8)  4,202 | 18 (0.2)  745 (8.1)  8,357 |
| BF 6 weeks/months  Missing outcome  Missing explanatory factors#  Complete case analysis | 48 (1.1)  335 (7.4)  4,126 | 34 (0.7)  395 (8.6)  4,182 | 82 (0.9)  730 (8.0)  8,308 |
| EBF 6 weeks/months  Missing outcome  Missing explanatory factors#  Complete case analysis | 239 (5.3)  303 (6.7)  3,967 | 211 (4.6)  345 (7.5)  4,055 | 450 (4.9)  648 (7.1)  8,022 |

#These numbers show those with any missing explanatory factors, after excluding those with missing outcome data
