# Supplementary material for: Breastfeeding rates in England during the Covid-19 pandemic and the previous decade: Analysis of national surveys and routine data
Source: PLoS One. 2023 Oct 11;18(10):e0291907. doi: 10.1371/journal.pone.0291907 (PMC10566678; doi:10.1371/journal.pone.0291907)
Supplement: S2 Table — (DOCX) [file pone.0291907.s007.docx]

**S2 Table**

**P-values for tests of interaction between survey year and each explanatory factor in turn**

|  | Initiation | BF 6 wks | BF 6 mths | EBF 6 wks | EBF 6 mths |
| --- | --- | --- | --- | --- | --- |
| Age | 0.55 | 0.76 | 0.20 | 0.62 | 0.60 |
| Education | 0.55 | 0.33 | 0.89 | 0.41 | 0.25 |
| IMD | 0.069 | 0.26 | 0.48 | 0.078 | 0.52 |
| Ethnicity | 0.82 | 0.76 | 0.56 | 0.78 | 0.43 |
| Country of birth | 0.56 | 0.41 | 0.61 | 0.66 | 0.47 |
| Parity | 0.105 | 0.41 | 0.47 | 0.44 | 0.31 |
| Caesarean section | 0.045 | 0.90 | 0.27 | 0.33 | 0.84 |
| Preterm birth | 0.30 | 0.098 | 0.066 | 0.12 | 0.24 |

Tests were performed using the final models as shown in Figure 3.
